# The Potential of High-Anthocyanin Purple Rice as a Functional Ingredient in Human Health

**DOI:** 10.3390/antiox10060833

**Published:** 2021-05-24

**Authors:** Supapohn Yamuangmorn, Chanakan Prom-u-Thai

**Affiliations:** 1 Lanna Rice Research Center, Chiang Mai University, Chiang Mai 50200, Thailand; supapohn.y@cmu.ac.th; 2 Agronomy Division, Department of Plant and Soil Sciences, Faculty of Agriculture, Chiang Mai University, Chiang Mai 50200, Thailand

**Keywords:** black rice, pigmented rice, rice anthocyanin, rice antioxidant

## Abstract

Purple rice is recognized as a source of natural anthocyanin compounds among health-conscious consumers who employ rice as their staple food. Anthocyanin is one of the major antioxidant compounds that protect against the reactive oxygen species (ROS) that cause cellular damage in plants and animals, including humans. The physiological role of anthocyanin in plants is not fully understood, but the benefits to human health are apparent against both chronic and non-chronic diseases. This review focuses on anthocyanin synthesis and accumulation in the whole plant of purple rice, from cultivation to the processed end products. The anthocyanin content in purple rice varies due to many factors, including genotype, cultivation, and management as well as post-harvest processing. The cultivation method strongly influences anthocyanin content in rice plants; water conditions, light quantity and quality, and available nutrients in the soil are important factors, while the low stability of anthocyanins means that they can be dramatically degraded under high-temperature conditions. The application of purple rice anthocyanins has been developed in both functional food and other purposes. To maximize the benefits of purple rice to human health, understanding the factors influencing anthocyanin synthesis and accumulation during the entire process from cultivation to product development can be a path for success.

## 1. Introduction

Anthocyanins have been demonstrated to reduce the risks of serious diseases such as cancer and obesity, and the compounds have antiviral, anti-inflammatory, and anti-skin aging effects [1,2,3,4,5,6]. In plants, although their function is not completely understood, the major role of anthocyanins has been reported as protecting against free radicals during physiological metabolism; in particular, anthocyanin can alleviate cell damage when plants are grown under biotic and abiotic stresses [7]. Therefore, consumption of naturally synthesized anthocyanin from plants for the benefit of human health can help reduce the risk of several major diseases.

**Figure 1:**
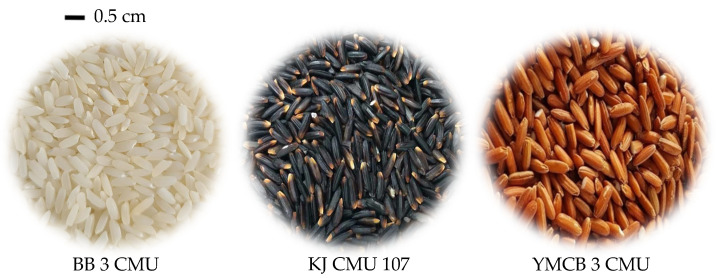
Characteristic rice kernels with non-pericarp color (BB 3 CMU), purple (KJ CMU 107) and red (YMCB 3 CMU) pericarp color.

Rice is one of the world’s major staple food crops, consumed by more than half of the world’s population along with crops such as wheat, maize, and potato; over 90% of the rice production areas and consumption amounts are recorded in Asia [8,9]. At least 175 countries and territories consume rice; the overall consumption is high in the rice-consuming countries, ranging from 100 to 200 kg of paddy rice per person per year according to the FAO. This could be one of the reasons why many international programs aimed at boosting human nutrition, e.g., the harvest plus biofortification with high zinc, iron, iodine, and selenium, are focused on rice crops [10]. Moreover, it is interesting to observe that among the staple food crops, rice is recognized as potentially containing high amounts of antioxidant compounds such as anthocyanin, especially in pigmented rice varieties with black (purple) and red pericarp color [11] (Figure 1). The purple rice is highly valued, particularly among the health-conscious consumers as a functional compound for human health among everyday sources of carbohydrate [12]. In other staple food crops, anthocyanin has been reported in purple maize, a variety that is rarely consumed in comparison with other non-staple food crops such as Brassica, Solanaceous species, and some edible flowers [13,14,15,16]. Additionally, anthocyanin can be directly taken from the concentrate in capsules for convenience, but this can be quite expensive [17,18]. Therefore, purple rice is an advantageous option for anthocyanin intake, especially when dealing with a large number of rice consumers worldwide.

The anthocyanin-rich rice grains have been recognized as excellent sources of natural and safe food colorants [19,20,21]. Synthetic colorants can be harmful to humans and the environment via allergic and toxic reactions [22]; this can raise the demand for naturally pigmented rice such as purple rice that contains high concentrations of anthocyanin. Thus, purple rice not only provides enough anthocyanin to fulfill the growing interest but also can increase the value of rice products. There are numerous rice varieties containing anthocyanins, but only some are accepted as commercial varieties due to yield potential, cooking quality, and other functional properties that may be less acceptable. This review will focus on the purple rice market trend, the possible sources of anthocyanin from different plant parts of purple rice, the significant role of anthocyanin in plants and humans, and factors controlling anthocyanin in rice plants. We also examine the role of anthocyanin biosynthesis genes in the regulation of anthocyanin in rice, the stability of anthocyanin in rice and rice products, and the utilization of anthocyanin in purple rice for different purposes. Thus, this review should be useful for understanding the entire processes of purple rice production and postharvest management, from product processing to the benefits to consumers’ health.

## 2. Purple Rice’s Market and Trend

**Figure 2:**
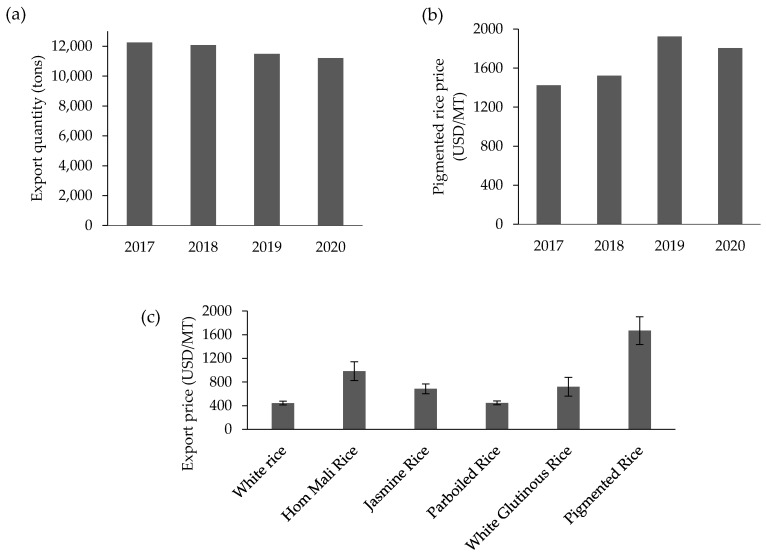
Thailand’s export quantity (**a**) and price (**b**) of pigmented rice and the average price of selected rice types (**c**) during 2017 to 2020. Source: drawn using data from OAE 2021.

Rice is the dominant cereal and staple food in many countries. Whilst non-pigmented rice (no pericarp color) is widely consumed throughout the world, pigmented rice varieties, e.g., purple rice, are also gaining interest in many regions. Purple rice is traditionally cultivated in Asian countries such as China, Japan, Korea, Thailand, Laos, Vietnam, Indonesia, India, Sri Lanka, and Nepal [23,24,25,26] as well as in other regions and countries including Brazil [27] In Asian regions, it has been established as a component of the traditional pharmacopoeia [28]. For example, traditional medicine in China uses pigmented rice to prevent anemia and to improve blood circulation, kidney function, and eyesight [29], while black porridge rice is given to aid recovery of broken bones [23]. Recently, Asian purple rice has attracted attention in the international rice market; at the same time, some new pigmented rice varieties have been bred and adapted to cultivation in other countries such as Italy, France, Russia, and Australia [30,31,32,33]. These events confirm that the worldwide demand for pigmented rice is increasing. Although Thai purple rice is currently produced on a very small scale compared with white rice, the export demand is expanding in various international markets. In fact, the export quantity has been fairly stable during 2017 to 2020, with an average of 11,762 tons annually, but the economic value has increased from 1423 to 1805 USD/MT according to the OAE (Figure 2a,b). Meanwhile, a higher market price trend for purple rice has been observed compared to other rice types including the premium fragrant rice type (Hom Mali rice) (Figure 2c) (computed from data in OAE, 2020) [34]. Additionally, the high value of purple rice has been largely provided by the online market, where the highest price for purple rice was recorded, especially organically produced rice with no chemical pesticides used (Siam Rice Export Company). Thus, the market trend for purple rice is heading in a good direction, especially when the production process can be precisely and safely controlled.

According to the increasing of demand for purple rice, meeting a quality standard has become necessary before exportation (Table 1) [35]. Purple rice typically has an intrinsic color from deep purple to black; the appearance and uniformity of the color are used to initially classify the quality grade (by eye) between the two different groups of endosperm type: non-glutinous and glutinous endosperm. This is because the non-glutinous purple rice is often required to have a higher standard level due to the greater demand compared with the glutinous purple rice. Although, regarding quality grade, there is currently not a significant gap indicating the difference in domestic market price, quality involves attractiveness as judged by customers. The color segregation is controlled by many factors during cultivation such as sunlight (quality and duration), day and night temperature, moisture content, rainfall, water conditions, and nutrient availability in the soil [36,37,38]. The shade and color uniformity of purple rice provide opportunities for competitive products in the health food market. However, unlike the common white rice, the price of quality grade purple rice for export has not been officially provided; discussing the reasonable value can encourage farmers and entrepreneurs to produce rice and rice products that reach maximum quality. Deeper studies of selection of rice varieties, practical management, and processing have implications on quantity and quality of purple rice, and this should be considered when the consumer’s interest is considered.

## 3. Anthocyanins in Different Plant Parts of Purple Rice

### 3.1. Variation in Grain Anthocyanin

**Figure 3:**
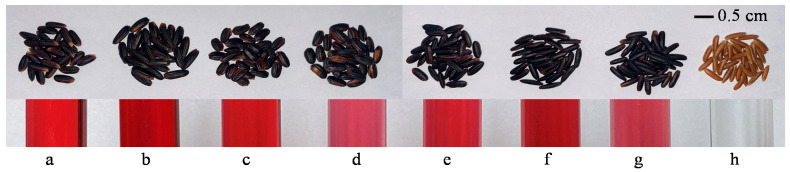
Examples of Thai purple rice varieties rich in anthocyanins. (**a**) Kum Doi Saket, (**b**) Kum Akha (**c**) Pieisu (**d**) Kum Hom CMU, (**e**) KJ CMU 107, (**f**) Riceberry, (**g**) Hom Nil, (**h**) Sang Yod.

Anthocyanins are localized in the outer grain layers, the so-called bran fraction, consisting of the pericarp, seed coat, nucellus, and aleurone layer [39]. Some anthocyanin is continuously distributed throughout the embryo, but it is rarely found in the endosperm [40], even though its accumulation in the endosperm occurs in the recently engineered purple rice [41,42]. The majority of grain anthocyanin is found in the pericarp and aleurone layers, comprising 85% of the whole grain content [43]. A study on the histological properties of rice confirmed that anthocyanins are mostly concentrated in the pericarp layer, especially in the dorsal side of the grain [44]. In general, anthocyanin concentration directly results from synthesis of the pigment responsible for the purple/black color [45]. Many researchers have demonstrated that rice grains consistently having dark colors usually contain high amounts of anthocyanin [46,47,48]. The variability in the visual grain color of the outer kernel and the colors of anthocyanin extracts of selected purple rice varieties are shown in Figure 3. We observed special cases such as Hom Nil and Riceberry that have similar color patterns but differ in how their colors of anthocyanin are extracted. Similarly, a variety with brown colored grains was classified into the highest anthocyanin group and exhibited an anthocyanin concentration higher than a fully dark variety [49]. A recent study also reported that anthocyanins could be combined with secondary structures of rice proteins by hydrophobic and hydrogen bonds [50]. This is interesting in that grain anthocyanin variability may depend on many factors such as variety, accumulation form, and location, all of which could be affected by environmental factors during cultivation practices.

Anthocyanin occurrence in the grain is very divergent, since most purple rice varieties are traditional/local rice that reflect differences among regions and growing environments (glutinous and non-glutinous rice types and upland and lowland rice types) [51,52]. Likewise, the majority of the genetic variation being within regions rather than between regions was reported in the traditional Philippine pigmented rice, and 589 pigmented rice accessions occur as both indica and japonica types [53]. In Northeastern Thailand, the total anthocyanin concentration of 30 local glutinous purple rice accessions ranged from 26 to 254 mg/100 g [54]. The latter study also evaluated total anthocyanin concentration among five glutinous rice varieties from Laos and found a wide range of concentrations, from 61 to 223 mg/100 g [55]. In other studies, a wide divergence in total anthocyanin concentration from 0 to 144 mg/100 g was found in whole grains of 29 Vietnamese purple rice accessions, and from 68 to 1730 mg/100 g in the bran of 25 Chinese purple rice varieties [47,49]. A recent report surveyed 30 glutinous pigmented rice varieties of the Chakhao landrace in India and found a genotypic diversity ranging from 30 to 276 mg/100 g [56]. Moreover, developing new varieties for a broad range of environmental adaptations and consumer purposes has led to a wide anthocyanin distribution. The variation of grain anthocyanin concentration in various rice varieties based on endosperm rice type according to ecotype, chemical properties, and quantification method is displayed in Table 2.

From the literature review, purple rice varieties cultivated in Asian countries are mostly found as local glutinous endosperm types, which overall have greater anthocyanin concentration compared to non-glutinous rice types. For example, the report of Pramai and Jiamyangyeun (2016) showed that eight glutinous rice samples across Thailand accumulated anthocyanins ranging from 81 to 442 mg/100 g, while a range from 21 to 85 mg/100 g was found among five non-glutinous rice samples [57]. Similarly, anthocyanin concentrations ranging from 42 to 271 mg/100 g and 12 to 40 mg/100 g were reported in four glutinous and four non-glutinous rice varieties, respectively [62]. In Indonesian rice, anthocyanin concentrations of glutinous rice samples ranging from 95 to 202 mg/100 g were higher than in non-glutinous rice ranging from 66 to 114 mg/100 g [66]. Additionally, exploring 36 purple rice accessions in Vietnam showed that most varieties classified in the high-anthocyanin group were glutinous rice [49]. These results suggest that glutinous purple rice would be representative of rice germplasm as a good source of materials for breeding programs designed to yield high anthocyanin rice grains. In recent years, a challenge for breeders has been to develop new varieties of non-glutinous purple rice with high anthocyanin stability based on selection of glutinous purple rice varieties [67,68]. Although the difference in anthocyanins between lowland rice and upland rice was not significant based on this review, several reports have demonstrated the performance of upland rice as a good source of anthocyanins and nutritional quality [36,69]. According to a recent review, southeast Asian countries such as Thailand, Laos, Vietnam, Indonesia, and the Philippines are sources of good purple rice accessions; however, there are few commercial rice varieties cultivated in these countries. Therefore, a study of genetic variability in local varieties related with anthocyanin pigmentation and other desirable characteristics will provide alternatives for rice farmers. When new varieties have been successfully developed to be more suitable to the environmental conditions and able to be cultivated in western countries, these varieties have been found to contain high grain anthocyanin concentrations (81 to 347 mg/100 g) [31,70].

Anthocyanin profiles have been extensively identified in rice grains, and these have been complex and divergent. Among the anthocyanins accumulated in rice bran of 25 purple rice varieties, cyanidin-3-glucoside comprised the highest proportion among all anthocyanins accounting for 82.3%, followed by peonidin-3-glucoside (14.6%), cyanidin-3-galactoside (1.2%), cyanidin-3-rutinoside (1.0%), cyanidin (0.7%) and peonidin (0.2%) [47]. Jiamyangyuen et al., 2019 identified cyanidin-diglucoside, cyanidin-3-sambubioside, cyanidin-3-rutinoside, peonidin3-rutinoside and pelargonidin-3-glucoside in Thai purple rice flour, but the levels were quite low [71]. Furthermore, the free and bound extracts showed differences in anthocyanin profiles; most of the cyanidin-3-glucoside and peonidin 3-glucoside was distributed in free form, but other anthocyanins such as cyanidin 3-galactoside, delphinidin, and cyanidin were found only in bound form [72]. A selection of the current data of anthocyanin profiles in various purple rice varieties is provided in Table 3. Many reports have shown the dominant anthocyanin in the grain was cyanidin-3-glucoside, followed by peonidin-3-glucoside. For example, the whole grains of cv. Kum Doi Saket showed similar proportions between the two anthocyanins, whereas rice bran of cv. Hom Nil had higher levels of peonidin-3-glucoside than cyanidin-3-glucoside [73,74]. Another study also found a higher proportion of peonidin-3-glucoside in the polished grains of an engineered rice variety (PER-Z#3) [42]. The variability in anthocyanin profiles from these samples may be due to differences in rice variety, anthocyanin localization, the nature of the sample, the measurement method, or the growing area. Since different anthocyanin profiles may result in differences in biological function and stability, the occurrence of each anthocyanin may be a significant trait indicating the potential application of rice varieties. Cyanidin-3-glucoside isolated from purple rice had greater ability to inhibit tumor cell growth and combat diabetes complications compared with peonidin-3-glucoside [75,76], but had a higher loss rate than peonidin-3-glucoside after being cooked in some rice samples [31]. This suggests that anthocyanin identification will facilitate understanding of the biological properties and stability of the anthocyanins in each rice variety. Many studies have determined the quantity and quality of the anthocyanins, but there is no clear grouping as to function. To date, genetic resources exhibiting high anthocyanin while demonstrating eating quality should be considered for specific purposes such as pharmaceutical products and natural colorants.

### 3.2. Occurrence of Anthocyanin in Vegetative Plant Parts

**Figure 4:**
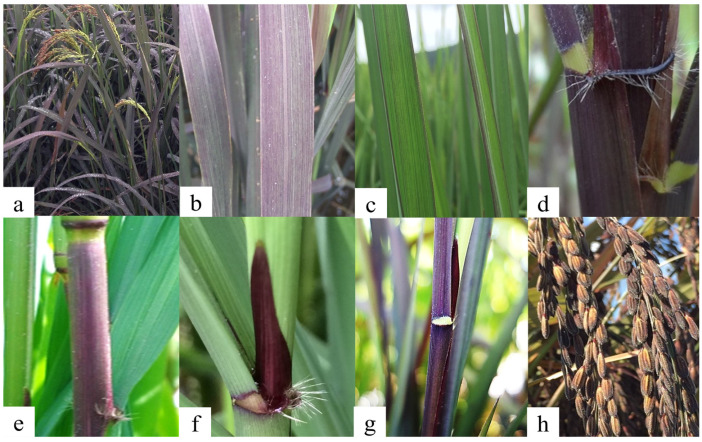
Anthocyanin pigmentation in the different plant parts. (**a**) Whole rice plant, (**b**) fully purple leaf blade, (**c**) margin purple leaf blade, (**d**) auricle, (**e**) node and internode, (**f**) ligule, (**g**) leaf sheath and (**h**) husk.

Anthocyanin in vegetative parts is mainly located in the vacuoles of upper and lower epidermis cells [80]. The anthocyanin can be visually observed in many parts of the plant, as the purple pigment is distributed in nodes, internodes, leaf sheaths, leaf blades, ligules, auricles, culms, and husks (Figure 4). The presence of a purple color in the vegetative part is a common phenotype in local rice; however, not all genotypes have the purple coloring in both shoot and grain [37]. A classification of fifteen purple rice varieties using morphological characters delineated two main groups: group 1 included varieties presenting purple anthocyanin vegetative tissues, and group 2 showed green or white characteristics [81]. Similarly, the characterizing by morphological traits of genotypic variation in anthocyanin in the purple leaf sheath was evaluated among 53 accessions, with 39 accessions possessing a green leaf sheath and 14 accessions possessing a sheath ranging from light to dark purple [82]. Although anthocyanins in vegetative parts have not been as often utilized as in the grain, the core roles of anthocyanin as a strong antioxidant to scavenge reactive oxygen species (ROS) and in tissue maintenance have resulted in the development of rice varieties with purple leaves [80,83,84,85,86].

As with anthocyanin in rice grains, the anthocyanins distributed in vegetative parts of purple rice plants depend on rice variety and plant part. The variation in total anthocyanin concentration has been evaluated among four local purple rice varieties (all varieties with purple coloration in nearly the whole plant) as ranging from 170 to 210 mg/100 g in leaf blades and from 67 to 100 mg/100 g in stem + leaf sheath; however, the concentrations declined with plant age across all four rice varieties [38]. In another variety that presented purple pigmentation in the leaf sheath, cyanidin-3-glucoside was identified using HPLC as the dominant anthocyanin (90%) at about 5.5 mg/100 g, while peonidin-3-glucoside was a minor component (10%) [87]. However, anthocyanin profiles from fully purple leaves in various rice varieties have been identified as containing much less in comparison to rice kernels. Moreover, rice husk anthocyanin concentration has been evaluated. Jha et al., 2017 were able to extract total anthocyanins from purple rice husk of cv. Poireton, yielding a total anthocyanin content of 3.39 mg/100 g [88]. In recent years, anthocyanin profiles have been characterized, and these have at times shown surprising concentrations. Anthocyanin in the purple rice husk accumulated cyanidin-3-glucoside as the major anthocyanin at up to 280 mg/100 g, while brown and straw-white husks contained none, in contrast to the concentration of delphinidin 3-rutinoside that was highly accumulated in brown husks and was higher than in purple husks [89]. In contrast, the anthocyanin profiles were dissimilar among other varieties and samples. A recent report showed that purple rice husk of cv. Kum Doi Saket contained malvidin-3-glucoside (71 mg/100 g) as the main anthocyanin, followed by cyanidin and peonidin-3-glucoside (55–56 mg/100 g), cyanidin-3-glucoside (32 mg/100 g), and small amounts of delphinidin-3-glucoside and peonidin (8 mg/100 g) [90]. In another sample of rice variety cv. Kum Doi Saket, cyanidin-3-glucoside was identified as the main anthocyanin, with a concentration of 196 mg/100 g, followed by peonidin-3-glucoside (115 mg/100 g) [91]. It has been noted that purple rice straw and the husk of a purple rice variety with peculiar nutritional properties would be efficient sources of anthocyanin for yielding both economic and environment benefits, and thus anthocyanin content should be screened among the various rice varieties.

## 4. Anthocyanin Biosynthesis Genes in Regulation of Anthocyanin in Rice

Genetic regulation is the first level describing expression of the structural and regulatory biosynthetic genes at which induction or inhibition of anthocyanin biosynthesis occurs in plants. Anthocyanin in rice grain has been well described in previous studies. Structural genes including phenylalanine ammonia lyase (PAL), cinnimate 4-hydroxylase (C4H), chalcone synthase (CHS), chalcone isomerase (CHI), flavanone 3-hydroxylase (F3H), flavonoid 3’-hydroxylase (F3’H), dihydroflavonol 4-reductase (DFR), and anthocyanidin synthase (ANS), anthocyanidin synthase (ANS), and UDP-glucosyl transferase (UGT) are involved in the anthocyanin biosynthesis pathway [92,93]. The transcription levels of purple rice cv. Chinakuromai observed in the caryopsis during grain filling were related with the expression level of *DFR*, which was highest followed by *ANS, LAR, CHS* and *CHI* in order [94]. In addition, Nayeem et al., 2020 recently studied the expression of eight structural genes in purple rice cv. Navara and showed that the transcription levels of *CHS, CHI, F3H, DFR, LAR* and *ANS* were maximal in the root, while only *PAL* was found in the stem [95]. The differences in the expression levels among genes may be due to the presenting of the various colors occurring in plants throughout the plant life cycle. However, the different gene expression levels related with anthocyanin occurrence have barely been studied in various purple rice varieties. Furthermore, the regulatory genes encoding transcription factors that modulate the expression of the structural genes have been widely studied in grain pericarp, while only recently have similar studies been done in the vegetative parts. Anthocyanin in rice grain is regulated by two families of bHLH genes (*Ra1*, *OsB1, Rb, Ra2 and OsB2*), and the R2R3-MYB gene (*OsC1*) [93,96,97]. Kim et al., 2018 also demonstrated that the high expression levels of *OsHY5*, *OsBBX14* and *OsB2* during seed maturation are associated with anthocyanin pigmentation in the grain [98]. To date, purple rice varieties with purple vegetative parts have seen increased interest concerning anthocyanin biosynthesis. A candidate regulatory gene, *plr4,* has been reported to be involved in pigment accumulation in rice leaves [84]. Hu et al., 2020 demonstrated that the combined effect of two regulatory genes consisting of the MYB (*OsC1*) and bHLH (*Rb1* and *Rb2*) loci resulted in substantial accumulation of cyanidin-3-glucoside and peonidin-3-glucoside in the leaf sheath [99].

In general, the pigmentation of anthocyanins can be fully functional depending on the coordination between the structural genes and regulatory genes. It has been found that the coordinated functioning of three genes comprising two structural genes, *OsDFR* and *ZmC1*, and one regulatory gene, *OsB2,* was involved in anthocyanin pigmentation in the leaf sheath [88]. In another study, the expression of a candidate regulatory *OsPA* gene in mutant rice resulted in anthocyanin coloration in the apiculus by upregulating the expression of the anthocyanin regulatory MYB gene (*OsC*) and the anthocyanin structural genes (*OsF3′H, OsF3′5 ′H, OsDFR*, and *OsANS*) [100]. Recently, Hu et al., 2020 demonstrated that the combined effect of two regulatory genes, the MYB (*OsC1*) and bHLH (*Rb1* and *Rb2*) genes, resulted in substantial accumulation of cyanidin-3-glucoside and peonidin-3-glucoside in the leaf sheath [99]. Moreover, anthocyanin pigmentation in purple rice husk has also been studied, and it has been reported that the expression of the DFR gene is the key step in the occurrence of cyanidin-3-glucoside, while loss of expression of this gene led to synthesis of other flavonoid compounds, resulting in a brown color in the husk [89]. To date, anthocyanin pigmentation in both vegetative parts and grains has not been successfully achieved. Although Akhter et al., 2019 showed that the collaboration between the structural genes *OsPAL*, *OsCHS*, and *OsANS* and the transcription factor gene *OsMYB55* can influence anthocyanin pigmentation in the leaf and leaf sheath of mutant purple rice plant, the anthocyanin coloration in rice pericarp and the yield were significantly affected [101]. Meanwhile, the regulatory genes *OsC1* and *OsRb* were identified as the determinants of anthocyanin biosynthesis in rice leaves, while neither gene functioned in the pericarp due to their being tissue-specific regulatory genes in vegetative parts [102]. Similarly, the structural genes *OsF3′H, OsDFR,* and *OsLDOX* are expressed in the vegetative plant parts, while grain anthocyanin was absent due to inhibition of the regulatory gene [103]. Thus, further research is required to unravel the mechanisms regulating gene expression in the vegetative parts as well as in the grain.

## 5. Genotype × Environment Interactions Controlling Anthocyanin in Rice

**Figure 5:**
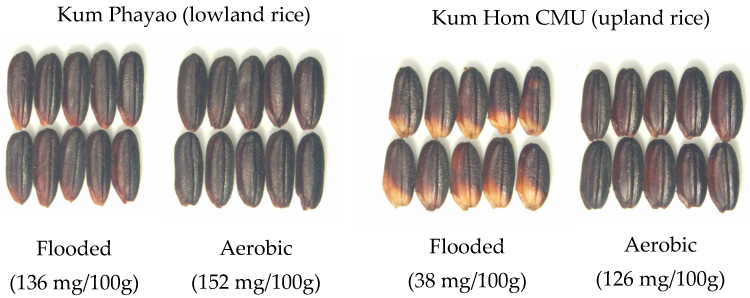
The responses of two purple rice varieties, cv. Kum Phayao (lowland rice) and Kum Hom CMU (upland rice), to flooded and aerobic soil conditions (the total anthocyanin concentrations are provided in the brackets).

The synthesis of anthocyanin in plants is controlled by an interaction between genetic and environmental factors. However, the optimum environmental conditions for maximizing anthocyanin accumulation in purple rice have rarely been studied. Water, light, temperature, and plant nutrients have been reported as environmental factors that affect anthocyanin accumulation in purple rice. Jaksomsak et al., 2020 showed that growing purple rice under flooded soil conditions resulted in higher anthocyanin accumulation, and a strong effect was found in upland rice compared with the lowland type [37]. The same study also demonstrated low anthocyanin concentration accompanied with grain discoloration in rice grown in flooded soil in comparison to aerobic soil, and this was similar in both wetland and upland rice varieties (Figure 5). These results indicated that the anthocyanin synthesis response to growing conditions in the water regime was independent of the rice variety, but it could also be related with the degree of water stress inducing anthocyanin synthesis.

Additionally, stimulation of anthocyanin synthesis can be induced by light and temperature. A study on anthocyanin accumulation in seven purple rice varieties reported that 50% shading level increased total anthocyanin concentration in all rice varieties from one to nine times higher than without shading, while the grain yield was reduced [104]. Furthermore, light quality affected anthocyanin formation in seedling leaves, as rice seedlings grown under red + blue LED had higher anthocyanin concentrations compared to seedlings grown under individual red or blue lights [105]. Temperature is another important environmental factor influencing grain anthocyanin synthesis. It has been reported that growing rice at the ambient temperature (27 °C) resulted in higher expression levels of anthocyanin biosynthetic genes during seed maturation related to the accumulation of cyanidin, cyanidin-3-glucoside, and peonidin-3-glucoside in purple rice grains, whereas gene expression was reduced at lower temperatures (21–24 °C) [92]. Another study on the effects of temperature during the grain filling stage and transplanting time using various rice varieties found that temperatures of 23–24 °C and a delayed transplanting time could increase total anthocyanin concentration and grain yield of purple rice, and that the greatest increase was observed in the early maturing varieties in comparison to middle and late maturing varieties [106]. However, the effect of temperature on the total anthocyanin concentration was not uniform between the leaf sheath and grain pericarp, as induction of low temperature (16 °C) at early tillering stage increased total anthocyanins in the leaf sheath compared with rice grown under ambient temperature (26–32 °C). However, the low temperature did not affect total anthocyanins in the grain pericarp [87]. In addition to environmental effects that can enhance anthocyanin accumulation, it is also necessary to consider their effects on grain yield. It has been reported that mycorrhizal fungi inoculation and magnesium spraying improved anthocyanin accumulation in rice grain and resulted in greater plant tolerance to cold and water deficit conditions [87,107]. While several environmental factors affecting anthocyanin synthesis in purple rice have been reported, the effect depends on the rice variety. Thus, it is difficult to specify the growing conditions that would reach the desirable anthocyanin accumulation in purple rice, as other environmental factors, e.g., soil fertility and nutrient management, also affect its synthesis and accumulation.

The enhancement of anthocyanin accumulation in purple rice has been achieved by fertilization. Yamuangmorn et al., 2018 reported that nitrogen fertilizer application increased anthocyanin in the leaf blade and stem + leaf sheath among four purple rice varieties. However, this did not significantly increase anthocyanin in the grain [38]. This illustrates that the biosynthesis pathway and accumulation mechanisms of anthocyanin could differ between the vegetative and reproductive organs; it would be interesting to investigate this point in future studies. In a recent report, the addition of mineral elements, especially calcium, strongly increased the total grain anthocyanin concentration by three-fold compared to the control, while selenium effectively increased anthocyanin in the leaf [108]. Previously, rice grain anthocyanin was induced by applying ZnO nanoparticles at 200 mg/L and was accompanied by an increase in enzyme antioxidant activity [109]. Moreover, supplementing the media with sucrose and nitrogen has been shown to increase flavonoid biosynthesis gene expression levels, resulting in accumulation of cyanidin and delphinidin in the rice callus. However, this effect needs to be confirmed in rice grain [110]. Although there are some studies reporting the enhancement of anthocyanin via manipulation of environmental factors, the responses of different experiments have been varied. Therefore, the study of interaction effects between rice variety and growing conditions on anthocyanin accumulation in purple rice will provide the necessary information on the selection of rice variety with appropriate growing condition to stabilize high anthocyanin content for improving plant and human health.

## 6. Stability of Grain Anthocyanin in Purple Rice during Post-Harvest Processing

Purple rice is harvested at maturity and is stored in the available conditions before processing for consumers. Post-harvest processing usually affects rice grain quality in both physical and chemical properties. Among many factors, temperature is critical for anthocyanin stability, as thermal conditions can cause de-glycosylation of anthocyanin molecules resulting in the loss of the B-ring and transformation to a colorless coumarin glucoside derivative [111]. Thermal drying is one method used to reduce moisture accumulation and the resulting anthocyanin degradation. Lang et al., 2020 reported that drying temperature increased degraded anthocyanin concentration, especially when the temperature reached 60 °C and above [112]. The loss of 50% of cyanidin-3-glucoside has been reported after hot air drying, but it has been established that drying with far infrared radiation can increase the anthocyanin content up to threefold, a result that can be explained by the effect of grain alerting leading to better yield extraction by thermal processes [113]. Thus, the effect requires further work to identify the physical and chemical reactions after treatment with the radiation, as alerting may not be sufficiently repeatable. In addition, purple rice anthocyanins were examined under different storage temperatures of 4 °C and room temperature, but no significant effect was obtained [114]. This result was in accordance with black rice grains stored at 16, 24, 32 and 40 °C for six months, where there was no significant difference in grain anthocyanins reduction [115]. In contrast, storage of brown rice grains in which the husk was removed and vacuum packaging under nitrogen-atmosphere has been shown to improve the reduction of anthocyanins during storage [112,114,116].

Cooking strongly affects grain anthocyanins [71,117]. In the laboratory, cooking purple rice in an autoclave resulted in a 56% reduction of anthocyanins [118]. However, the decreased content of anthocyanin via cooking can be avoided by using alternative methods. Using an electric rice cooker retained anthocyanins in glutinous rice better than using the pressure-cooker method [62]. Microwave cooking conserved two- to three-fold higher anthocyanin content in glutinous and non-glutinous rice in comparison to steam cooking [119]. For Italian purple rice, no anthocyanin reduction was obtained after cooking using either a rice cooker or a water bath [120]. Similarly, cooking with risotto showed stable cyanidin-3-glucoside content [31]. In addition to the thermal effect on anthocyanin reduction, anthocyanins are directly affected by soaking in water and increasing the soaking time compared to total cooking time. Cooking without soaking and/or a reduced soaking time prevented anthocyanins from leaching into the water [62]. Likewise, cooking by boiling with a low amount of water has been reported as an effective method of preserving anthocyanins; the effect resulted from the decrease in boiling time [121]. Thus, since the rice cooking methods are based on cultural practices according to rice type and the consumer’s taste, the maintenance of anthocyanins via thermal cooking is influenced by many factors. The most appropriate method should allow purple rice cooked grains to retain the highest anthocyanins content while yielding a desired taste.

Roasting rice grain is a traditional process performed on well-done cooked rice to improve aroma, flavor, and taste. However, grain anthocyanin stability is affected by roasting. A reduction of purple rice anthocyanins was obtained after microwave roasting [122]. Meanwhile, roasting at 100 °C for 20 min slightly increased anthocyanin content, but a reduction was observed in roasted grains at higher temperatures and longer roasting times [123]. In addition, other rice processing practices in the food industry such as drum drying, and extrusion could lead to anthocyanin loss [124]. From the literature review, every step-in post-harvest processing related to thermal temperature affected grain anthocyanin stability, and thus there are many factors that influence the final intake of anthocyanins among consumers.

## 7. Utilization of Anthocyanin in Purple Rice as Functional Compounds

The unique antioxidant properties of anthocyanins have led to various applications in areas from health foods to beauty products. Recently, the physical and chemical functions of anthocyanins extracted from rice have been used as biosensors and natural dyes. Anthocyanin applications can be divided into functional and other purposes (Table 4). Thus, anthocyanin stability is needed to investigate processes related with temperature, pH, solvent, and pretreatment [125,126,127]. Furthermore, advanced techniques such as microencapsulation that ensure minimal anthocyanin degradation in food products as well as the human digestive tract have been intensively investigated [17,18,128,129].

## 8. Significant Roles of Anthocyanin from Purple Rice in Human Health

Anthocyanin compounds are mediators of ROS, and as such are a unique characteristic of stressed plants. Therefore, most literature reviews tend to focus on the roles of anthocyanins in enhancing tolerance to abiotic and biotic stresses. Zaidi et al., 2019 showed that high temperature during grain development resulted in declines in grain weight and kernel starch content, and a mutant rice plant displaying grain anthocyanin production was much less damaged by high temperatures compared to wild-type plants without grain anthocyanin pigmentation [146]. In addition, anthocyanin has been used to distinguish drought-responsive traits. Tiwari et al., 2021 showed anthocyanin accumulation was induced in a drought-tolerant variety, and this was related with the increase in the transcription level of regulatory genes involved in antioxidative mechanisms [147]. Similarly, a previous study found that the purple rice variety that can produce anthocyanin in leaves at higher levels under drought conditions could alleviate the degree of drought damage [148]. Regarding the study of metabolite compounds of various purple rice varieties grown under dry-land cultivation, it has been demonstrated that anthocyanins could maintain energy metabolism, carbon assimilation, and the transduction of stress signals to support starch accumulation [149]. This accords with a previous study by Chunthaburee et al., 2016 who showed that purple rice plants having a deep purple leaf color were less affected by salt stress than plants with a greenish purple leaf color due to higher anthocyanin concentration as well as higher levels of anthocyanin biosynthesis gene expression [150]. Besides protective effects during plant growth, anthocyanins may also play important roles in controlling pests and maintaining plant growth. Several recent studies have reported that the increased expression levels of structural anthocyanin genes related with anthocyanin in rice plants are believed to be significant traits for enhancement of rice resistance to the white-backed planthopper [151,152]. A strong positive relationship between anthocyanin concentration and seedling vigor in rice has been also reported [153]. These studies indicate that anthocyanins are required for rice plants to survive in stressful environments. However, the case of purple rice varieties having natural anthocyanin synthesis in vegetative organs has not yet been compared with greenish plants regarding the alleviation of growth resulting from increased anthocyanin synthesis. This would provide strong evidence to support the essential roles of anthocyanins in purple rice plants and thus should be considered and evaluated in various environments.

While it is well documented that anthocyanin is essential in anti-oxidative functions of plants, numerous studies have suggested that anthocyanins from purple rice have other biological activities, and these may be positively associated with human health. The benefits of purple rice anthocyanins have been tested via both in vitro and in vivo studies. Recently, in vitro studies have reported that anthocyanins reduce the risk of obesity by lowering glucose uptake and inhibiting adipocyte formation and proliferation [4]. Similarly, purple rice extract affected reduction of carbohydrate and lipid digestion and absorption in Caco-2 cells [154]. In addition, the purple rice anthocyanins have been shown to exhibit anti-inflammatory effects in IL-1β-stimulated human chondrocytes as well as exert an anti-metastatic effect on HER-2-positive breast cancer cells [1,6]. Furthermore, anthocyanin from purple rice has been reported as an efficient compound in pharmaceutical production. The cosmetic industry has recognized the value of inhibition of ROS generation in anti-aging products, where purple rice anthocyanins can be used as cosmetic ingredients for preventing skin photoaging [155,156]. Recently, food bioactive compounds have been highlighted for supporting the immune system against COVID-19 [157]. Likewise, anthocyanins have been recommended compounds for significant antiviral enhancement, and they have recently been reported to act against the protease enzymes of COVID-19 [2,3,158]. The established evidence has clearly confirmed the positive effects of purple rice anthocyanins, while studies using in vivo models have been increasingly employed to examine the effects on various diseases.

In the in vivo studies, anti-hypoglycemic and anti-osteoporosis effects of purified cyanidin-3-glucoside have been demonstrated in the kidneys; the effects are produced by reducing blood glucose and by suppressing oxidative stress and inflammation [159]. Additionally, anthocyanin in rice starch has inhibited the activities of starch digestive enzymes resulting in the reduction of blood glucose levels in mice, thereby positively affecting diabetes mellitus [160]. A significant body of evidence has supported the preventive efficacy of cyanidin-3-glucoside isolated from purple rice in suppression of allergic airway inflammation in lung tissues [161]. The alleviation of the symptoms and inflammation of colitis was demonstrated in dietary purple rice anthocyanin-rich extracts [162]. Additionally, the extracted anthocyanin from purple rice grain has been suggested to reduce gastroduodenal symptoms caused by microbial infection as well as improve cholesterol metabolism and gut microbiota dysbiosis in high-fat and high cholesterol diet groups [163,164]. Recently, the benefits of purple rice rich in anthocyanins in boosting immune responses have been shown to function by increasing the numbers of immune cells such as white blood cells, lymphocytes, and neutrophils [165]. A previous study indicated that the risk of leukemia was reduced after intake of purple rice anthocyanin by increasing the population of white blood cells and promoting macrophage phagocytic activity [166]. Moreover, the anti-inflammatory properties of anthocyanin extracted from purple rice have been investigated in the treatments of wound healing of psoriasis and oral mucositis in animal models [167,168]. Most recently, anthocyanins isolated from rice husk have been demonstrated to be novel anticarcinogenic compounds in the liver due to their cancer chemo-preventive properties [90].

According to reports concerning the role of anthocyanins in the prevention and treatment of disease, the consumption of anthocyanin from purple rice has been confirmed to benefit human health. This includes research that identified consumption of bread made from anthocyanin-rich purple rice by healthy participants resulting in lower postprandial plasma glucose concentration and postprandial plasma insulin compared with consumption of white rice. Meanwhile, anthocyanin consumption also improved the antioxidant status of the plasma [130]. However, the effects of anthocyanins on health have not been well studied in humans, suggesting that the study of suitable anthocyanin dosage is a serious topic that needs to be examined in the future. The above literature review found that recommended purple rice anthocyanin extract dosages estimated from animal models had a wide range that may be due to the differences in sample extracts, target diseases, and participants. For example, dosages from 95 to 190 mg per day for a person weighing 60 kg were recommended for diabetes diet control [159]. A supplement with 400 mg of purified cyanidin-3-glucoside per day for a 60 kg adult effectively inhibited airway inflammation [161]. The dose of 100 mg/kg of body weight per day of purple rice anthocyanin-rich extract was suggested in the treatment of inflammatory bowel diseases [162], while the daily intake of 1000 mg/kg of body weight had more effectiveness on immunity enhancement when compared with 100 or 300 mg/kg [165]. In addition, Wang et al., 2020 reported that 5.84 mg/kg body weight/day (equal to 350 mg/day for a person weighing 60 kg) of purple rice anthocyanin extract could protect against liver steatosis in high-fat and high cholesterol diet groups [164]. Although the daily intake of purple rice anthocyanins can be estimated from the supplements in animal studies, it has not yet been shown in human models to be related with bioavailability and metabolites of anthocyanins. The appropriate dosage of the extracted anthocyanins from purple rice should be carefully evaluated using the covariates of gender, age, weight, and health condition; otherwise, the whole purple rice grain can be consumed to avoid overdoses.

## 9. Conclusions

Purple rice rich in anthocyanins is widely consumed directly as a food and indirectly as an ingredient in alternative products. The consumer’s demand for healthy foods is driving purple rice to be more visible and acceptable as a potential natural source of anthocyanin. The benefits of anthocyanin in human health have been intensively investigated via both in vivo and in vitro models of chronic diseases. The conclusions obtained from this review article confirm the results of recent research that has focused on the effects of anthocyanin on non-chronic diseases. This knowledge can be used as a guide for how anthocyanin-rich purple rice affects human health. However, it is necessary to consider the bioavailability and metabolites of anthocyanins in human physiology. This would provide daily intake amounts of anthocyanin according to gender, age, weight, and health condition. In addition, the strong anti-inflammatory effect and antioxidant activity of anthocyanins have resulted in the production of novel bioactive compounds in non-food goods such as cosmetics and skin care products. However, the intake of anthocyanins by direct grain and the utilized form should be evaluated for both farmers and entrepreneurs. At the same time, it will be interesting to investigate other sources of anthocyanins from the by-products of purple rice processing such as defatted rice bran, husk, and straw, as these can add the value to less economically important biomaterials as functional ingredients and natural dyes. Therefore, the performance of purple rice cultivation and optimal post-harvest processing related to anthocyanin’s yield and properties should be involved in determining the final uses for maximizing the potential of anthocyanins from purple rice.

## Figures and Tables

**Table 1:** Thailand’s export quality standard specifications for pigmented rice.

Grade	Entirely Dark or Almost Entirely Dark Kernels (%)	Allowed Off Color Kernels (%)	Other Color Kernels (%)	Other Type Kernels (%)	Size of Head Rice (Part) (mm)
Non-glutinous rice					
Prime quality	≥80	≤20	≤4.0	≤1.0	≥7
Superb quality	≥65	≤35	≤4.0	≤1.0	≥7
Glutinous rice					
Best quality	≥30	≤70	≤6	≤1.0	≥6.5
Standard quality	≥15–<30	>70–≤ 85	≤6	≤1.0	≥6.5

Source: DFT, 2019.

**Table 2:** Total anthocyanin concentration (TAC) and some chemical properties of purple rice varieties across two endosperm rice types. The ecotype, nature of rice sample, and measurement method are also listed.

Variety	Ecotype	TAC (mg /100 g)	Amylose (%)	Aroma Property	Rice Sample	Measurement/Extract Solution	Reference
Glutinous Rice							
Niaw Dam Pleuk Dam	unknown	381	9.7	nd	whole grain	pH different/acidified methanol	[57,58]
Niaw Dam Pleuk Khow	lowland	442	8.9	nd	whole grain	pH different/acidified methanol	[57,58]
Kum Doi Saket	lowland	119	6.2	A	whole grain	pH different/ acidified methanol	[59,60]
Leum pua	upland	212	3.4	A	flour	pH different/nd	[60,61]
Pieisu	upland	271	nd	nd	whole grain	pH different/acidified methanol	[62]
KHCMU	upland	58	nd	A	whole grain	pH different/acidified methanol	[62]
Kam Med-dam	upland	222	nd	nd	whole grain	pH different/acidified methanol	[55]
Kam Leuang-dam	upland	150	nd	nd	whole grain	pH different/ acidified methanol	[55]
Kalobhat	unknown	57	5.4	nd	whole grain	Colorimetric/acidified methanol	[24]
Chakhao	lowland	276	6.0	A	flour	HPLC/acidified methanol	[56]
Non-Glutinous Rice							
Hom Nil	lowland	65	14.2	nd	whole grain	Colorimetric/distilled water	[63]
Riceberry	lowland	129	17.5	nd	flour	pH different/nd	[62]
Malinil Surin	lowland	75	13.8	nd	flour	pH different/nd	[60]
KJ CMU 107	lowland	12	nd	A	whole grain	pH different/acidified methanol	[62]
Mamihunger	unknown	34	17.6	nd	whole grain	colorimetric/ acidified methanol	[24]
Italian rice	lowland	54	9.9	nd	whole grain	pH different /acidified methanol	[64]
Jatinangor	unknown	40	nd	nd	Whole grain	colorimetric/ethanol and citric acid	[65]

nd means not detected. A = contains aroma property.

**Table 3:** Major anthocyanin species (cyanidin-3-glucoside; C3G and peonidin-3-glucoside; P3G) in various purple rice varieties.

Variety	Rice Sample	Concentration (mg/100 g)		Ref.
		C3G	P3G	
Kum Doi Saket CMU 125	whole grain whole grain	50 200	48 65	[73]
Hom Nil Riceberry	whole grain whole grain	133 48	20 7	[74]
Hom Nil Leum Pua	Bran bran	926 2277	1422 792	[77]
WC 320	bran	1103	33	[47]
Khao Gam Pah E-Kaw	whole grain	88	32	[78]
Artemide	whole grain	199	11	[31]
Chakhao	whole grain	208	45	[56]
IAC 600	whole grain	130	10	[27]
Heimi 2420	whole grain	111	31	[79]
PER-Z#3 PER-Z#14	polished grain polished grain	53 27	44 26	[42]

**Table 4:** Different applications of purple rice anthocyanins in functional food and other purposes.

Purple Rice Anthocyanin Application	Ref.
Functional Food	
Bread made from anthocyanin-rich purple rice improved postprandial plasma glucose and antioxidant status in healthy subjects	[130]
Anthocyanin-rich purple rice flour can be used as a gluten-free ingredient in bread providing FRAP antioxidant activity	[131]
Bread fortified with 1–4% of anthocyanin-rich rice powder has a low digestion rate that provides health benefits	[132]
Anthocyanin-rich rice beverage added with xanthan gum has high thermal stability	[133]
Germinated purple rice that retains anthocyanins has good sensory characteristics	[134]
Crispy rice bar made purple rice provides high anthocyanin	[135]
Fresh germinated purple rice noodles provide total anthocyanin and DPPH and FRAP antioxidant capacity	[136]
15% of anthocyanin-rich purple rice extract supplemented pasta contains high anthocyanins and antioxidant capacity (DPPH and FRAP)	[137]
Purple rice sprouts present high total anthocyanin and could be developed for natural health products	[138]
0.25% of anthocyanin-rice purple rice extract supplemented yogurt suppresses postprandial glucose level and improved plasma antioxidant capacity in healthy volunteers	[19]
0.06% of purple rice anthocyanin extract inhibited lipid and protein oxidation in whey-protein-stabilized food emulsions	[21]
**Other Purposes**	
Anthocyanin extract used in cream exhibited in vitro antioxidant activity and in vivo anti-ageing activity on human skin	[139]
Rice bran extracts containing anthocyanins have been investigated as ingredients in the cosmetic formulations that exhibit antioxidant capacity	[140]
Purple rice anthocyanin is used in the colorimetric sensing of Al^+3^	[141]
Packaging film based on oxidized chitin nanocrystals/gelatin incorporating purple rice bran anthocyanins has potential in freshness monitoring	[142]
3% of purple rice bran anthocyanins added in oxidized-chitin nanocrystals/chitosan matrix are able to monitor the spoiling of seafoods	[143]
1% of purple rice anthocyanins incorporated into chitosan packing films can be used to monitor pork spoilage	[144]
Anthocyanin dye increased the performance of dye-sensitized solar cells	[145]
